# IBI: Identification of Biomarker Genes in Individual Tumor Samples

**DOI:** 10.3389/fgene.2019.01236

**Published:** 2019-11-26

**Authors:** Jie Li, Dong Wang, Yadong Wang

**Affiliations:** School of Computer Science and Technology, Harbin Institute of Technology, Harbin, China

**Keywords:** biomarker, individual sample, tumor, regression model, gene expression data

## Abstract

Individual patient biomarkers have an important role in personalized treatment. Although various high-throughput sequencing technologies are widely used in biological experiments, these are usually conducted only once or a few times for each patient, which makes it a challenging problem to identify biomarkers in individual patients. At present, there is a lack of effective methods to identify biomarkers in individual sample data. Here, we propose a novel method, IBI, to identify biomarkers in individual tumor samples. Experimental results from several tumor data sets showed that the proposed method could effectively find biomarker genes for individual patients, including common biomarkers related to the mechanisms of the development of cancer, which can be used to predict survival and drug response in patients. In summary, these results demonstrate that the proposed method offers a new perspective for analyzing individual samples.

## Introduction

Biomarker discovery is critical for cancer diagnostics, prognosis, and monitoring of therapy in clinical trials. With the development of high-throughput biochip technologies such as next-generation sequencing, massive quantities of cancer genomic data are being generated in the healthcare field, which offers an opportunity to identify high-quality cancer biomarkers for use in personalized medicine. Therefore, various computational methods have been proposed to identify cancer biomarkers. At present, the most commonly used methods are statistical tests, such as t-test, KS-test, and Wilcoxon’s rank sum test (Li et al., 2007; Dembélé and Kastner, 2014; Love et al., 2014; Moore et al., 2016; Wang et al., 2018), which identify differentially expressed genes (DEGs) from two types of samples and choose the group of genes with the lower p-value as potential biomarkers. However, the method often ignores and misses information between genes (Lewis-Wambi et al., 2008). Machine learning algorithms and statistical models also are widely used to identify cancer biomarkers. For example, the 70-gene biomarkers (Van’t Veer et al., 2002), wound-response gene biomarkers (Chang et al., 2005), and several of our gene biomarkers (Li et al., 2008; Li et al., 2010; Zhang et al., 2017) are all identified using machine learning algorithms. The 21-gene biomarkers (Van’t Veer and Bernards, 2008) and immunotherapy response biomarkers (Ock et al., 2017; Jiang et al., 2018) are based on statistical models.

However, the above methods are only able to identify biomarkers in two groups of samples, not in an individual sample. As cancer is a complex and heterogeneous disease, different patients have differences in pathogenesis and need different treatments. Thus, there is a need for biomarkers for individual patients that reflect their status. Currently, high-throughput biological experiments are usually conducted once or a few times for a single patient, which makes it a challenging problem to analyze single samples and, in particular, to identify biomarkers in individual patients. Some algorithms have been developed to analyze single samples. Rezwan et al. (2015) used the Crawford-Howell t-test to analyze methylation data of single samples and identified hypomethylation at different sites. However, this method could only detect differences in a single molecular element among different samples and may ignore the relationships of different molecular elements in the same sample. Liu et al. (2017) proposed the sDNB (single-sample dynamic network biomarkers) method to detect early-warning signals or critical states in individual patients using gene expression data. sDNB detects changes in gene expression levels of a pair of genes relative to reference samples and considers the local information of a gene in network. Drier et al. (2013) proposed an algorithm to analyze single tumor samples using pathway-level information instead of gene-level information. Pathways were detected that were significantly associated with survival of glioblastoma and colorectal cancer patients. However, a set of genes in the same pathway have similar functions; this means that models based on redundant features (biomarkers) are usually more complex.

**Figure 1:**
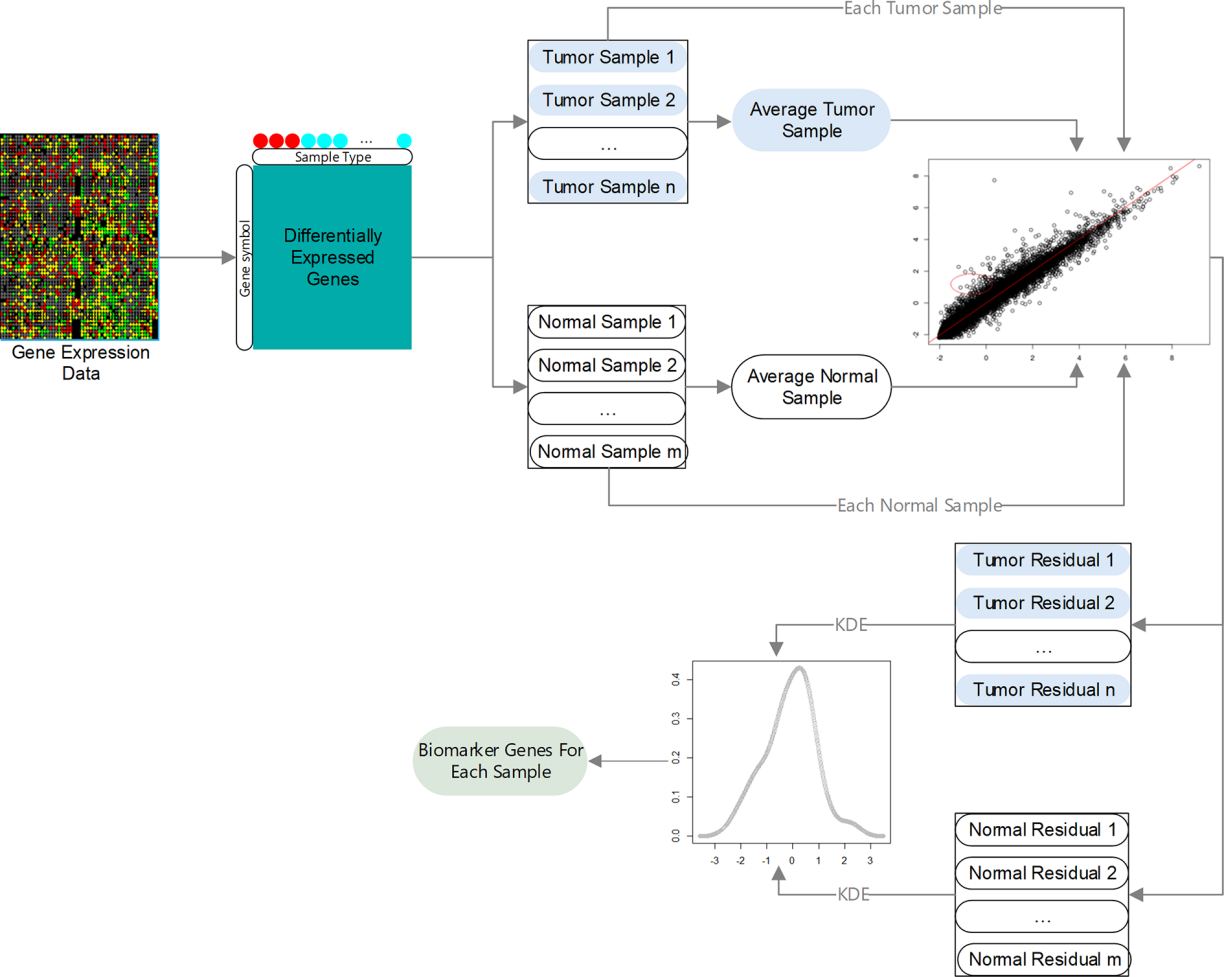
Overview of IBI method.

Here, we propose a novel method, IBI (identification of biomarker genes in individual tumor samples), to identify biomarker genes in individual tumor samples using gene expression data. An overview of the IBI method is given in **Figure 1**. First, DEGs in tumor and normal samples are identified. Then, regression models are constructed using the selected DEGs, and residuals of each gene in different samples are analyzed using the kernel density estimation (KDE). Finally, we assess the degree of change of each gene according to the credibility interval (CI) of its residuals to decide which genes are biomarkers of the individual sample.

## Materials and Methods

### Data Collection and Preprocessing

The proposed method was used to analyze three gene expression data sets: TCGA-BRCA (Tomczak et al., 2015), GSE63557 (Lesterhuis et al., 2015), and GSE35640 (Ulloa-Montoya et al., 2013). TCGA-BRCA consists of 1,090 breast cancer samples and 113 normal tissue samples. GSE63557 contains AB1-HA tumor data from mice during immunotherapy with 10 anti-CTLA-4 immunotherapeutic response samples and 10 non-response samples, and GSE35640 consists of advanced melanoma data with 22 MAGE−A3 immunotherapeutic response and 34 non-response samples. The first data set contains RNA-seq data, which was preprocessed using DESeq2 (Love et al., 2014), and the latter two data sets were preprocessed using the z-score.

### Identification of Differentially Expression Genes

Assuming we have gene expression data with two types of samples and genes, let each sample be labeled with either “+” or “−”; *n*_1_ and *n*_2_ are the number of samples with label “+” and “−”, respectively (*n* = *n*_1_+ *n*_2_). *y* *_ji_* is the expression value of the *j*th gene of the *i*th sample with label “+”, and *x* *_ji_* is the expression value of the *j*th gene of the *i*th sample with label “−”. *q* DEGs are obtained using the robust algorithm (Love et al., 2014) or GEO2R (Smyth, 2004).

### Average Sample

Let average samples with label “+” and “−” be $u^{+}=\left[u_{1}^{+},u_{2}^{+}\ldots u_{q}^{+}]\;and\;\;u^{-}=[u_{1}^{-},u_{2}^{-}\ldots u_{q}^{-}\right]$, respectively.

(1) $$\begin{array}{l} \begin{array}{cc} u_{j}^{+}=\frac{1}{n_{1}}\sum_{i=1}^{n_{1}}y_{ji}, & q\geq j\geq 1 \end{array} \end{array}$$

(2) $$\;\begin{array}{cc} u_{j}^{-}=\frac{1}{n_{2}}\sum_{i=1}^{n_{2}}x_{ji}, & q\geq j\geq 1 \end{array}$$

### Regression Model Based on Average and Single Samples

Let $\;y_{ji}^{\text{'}}$ be the expression value of the *j*th DEG of the *i*th sample with label “+” and $\;x_{ji}^{\text{'}}$ the expression value of the jth DEG of the *i*th sample with label “−.” For the *i*th sample with label “+,” $S_{i}^{+}=\left[{y^{\prime }}_{1i},\;{y^{\prime }}_{2i}\;.\;.\;.\;{y^{\prime }}_{qi},\right]$,  $\;{y^{\prime }}_{ji}$ can be predicted using the following regression model according to $u_{j}^{+}$:

(3) $$\begin{array}{cc} \;\hat{{y^{\prime }}_{ji}}=\beta_{0}^{+}+\beta_{1}^{+}u_{j}^{+}\;, & q\geq j\geq 1 \end{array}\;$$

where $\beta_{0}^{+}$ and $\beta_{1}^{+}$ are the regression coefficients estimated according to a set of data $\left(y_{1i},u_{1}^{+}\right)$, $\left(y_{2i},u_{2}^{+}\right)$ , …, $\left(y_{qi},u_{2}^{+}\right)$ , using the least squares method.

Similarity, for the *i*th sample with label “−” $,\;S_{i}^{-}=\left[x_{1i}^{\text{'}},x_{2i}^{\text{'}}\ldots x_{qi}^{\text{'}}\right]\;,$ $x_{ji}^{\text{'}}\;$ can be predicted using the following regression model according to $u_{j}^{-}$:

(4) $$\begin{array}{cc} \hat{x_{ji}^{'}}=\beta_{0}^{-}+\beta_{1}^{-}u_{j}^{-}\;, & q\geq j\geq 1 \end{array}\;$$

where $\beta_{0}^{-}$ and $\beta_{1}^{-}$ are the regression coefficients estimated according to a set of data $\left(x_{1i},u_{1}^{-}\right)$, $\left(x_{2i},u_{2}^{-}\right)$ , …, $\left(x_{qi},u_{q}^{-}\right)$ using the least squares method.

### Algorithm for Identifying Biomarker Genes of a Single Sample

Among *q* DEGs, expression values of some genes of a single sample may undergo very significant changes compared with their average values, i.e., the observed values of these genes are far from regression line. These genes are called biomarker genes of the single sample. The degree of the significant difference can be calculated using the residual value between the predicted value and observed value.

For the *i*th sample with label “+,” the residual value of its the *j*th DEG is:

(5) $$e_{ji}^{+}={y^{\prime }}_{ji}-\;\hat{{y^{\prime }}_{ji}}\;,\;\;\;\;\;\;\;q\geq j\geq 1$$

Similarity, for the *i*th sample with label “−”, the residual value of it’s the *j*th DEG is:

(6) $$e_{ji}^{-}=x_{ji}^{'}-\;\hat{x_{ji}^{'}}\;,\;\;\;\;\;\;\;q\geq j\geq 1$$

To obtain biomarker genes of the *i*th sample with label “+”, the KDE is introduced to estimate the probability density function $\;\hat{f_{i}^{}}\left(e_{i}\right)$ of residual values: $\left(e_{1i}^{+},e_{2i}^{+},\;...,\;e_{qi}^{+}\right)$. Its kernel density estimator with Gaussian kernel K is as follows:

(7) $$\hat{f_{i}^{}}\left(e_{i}\right)=\frac{1}{qh}\sum_{j=1}^{q}K\left(\frac{e_{i}-e_{ji}^{+}}{h}\right)$$

(8) $$K\left(x\right)=\frac{1}{\sqrt{2\pi }}e^{-\frac{1}{2}x^{2}}$$

where h is a smoothing parameter called the bandwidth (h > 0). Let Φ be the cumulative distribution function of the kernel density estimator; then, the CI at confidence level α is

(9) $$CI_{\alpha }=\left(0,\Phi \left(\frac{\alpha }{2}\right)\right)\cup^{\text{​}}\left(\Phi \left(1-\frac{\alpha }{2}\right),1\right)\;$$

The *j*th gene is considered a biomarker gene of the *i*th sample with label “+” (*n*_1_ ≥ *i*≥ 1) if $\Phi \left(e_{ji}^{+}\right)\in CI_{\alpha }$ . Similarity, we can obtain the biomarker gene of the *i*th sample with label “−”(*n*_2_ ≥*i* ≥1).

## Results

### Performance Evaluation

It was somewhat difficult to directly evaluate the performance of the proposed method. Three methods were employed to evaluate the power of the method.

1. Statistical test: The biomarker genes of each sample should be specific, that is, their expression values in the sample should be significantly different from those of other samples. We designed a method to test such differences, as follows. First, biomarker genes of sample *S* *_i_* are selected and their expression values extracted from all samples. Then, the expression values of each biomarker gene in different samples are sorted respectively and used to construct a rank matrix. The *i*th row vector, *R* *_i_*, of the matrix denotes orders of biomarker genes of *S* *_i_*. Finally, the Kolmogorov-Smirnov test is performed to determine whether there is a significant difference between *R* *_i_* and *R* *_j_* (*j*≠*i*).
2. Survival analysis: The biomarker genes of each tumor sample should reflect its characteristics, namely, it should be possible to use biomarker genes to classify tumor samples into high- and low-risk groups and predict the survival risk of tumor patients.
3. Validation *via* biological evidence: The biomarker genes of each tumor sample should reflect the pathogenesis of cancer, that is, they should have been reported to be associated with tumor development in the published literature.

### Experimental Results for TCGA-BRCA

**Figure 2:**
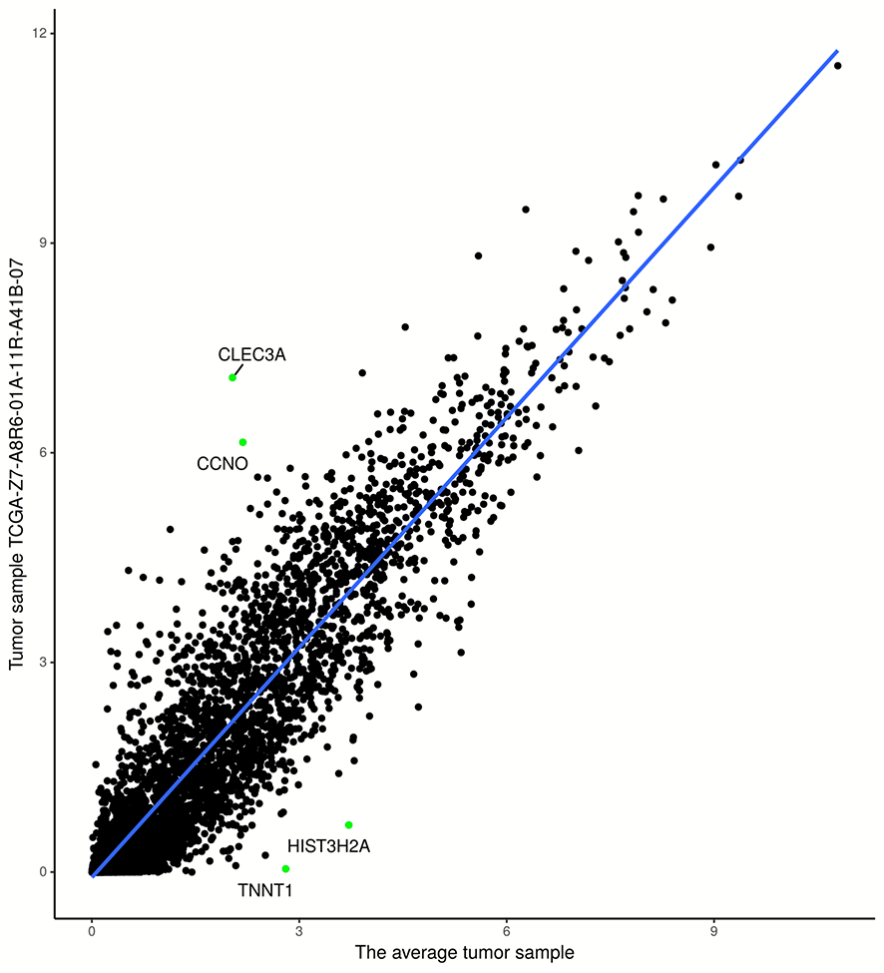
Regression model based on tumor sample TCGA-Z7-A8R6-01A-11R-A41B-07 and average tumor sample. The points in the upper-left (lower-right) partition are two biomarker genes with the highest (lowest) expression levels.

**Figure 3:**
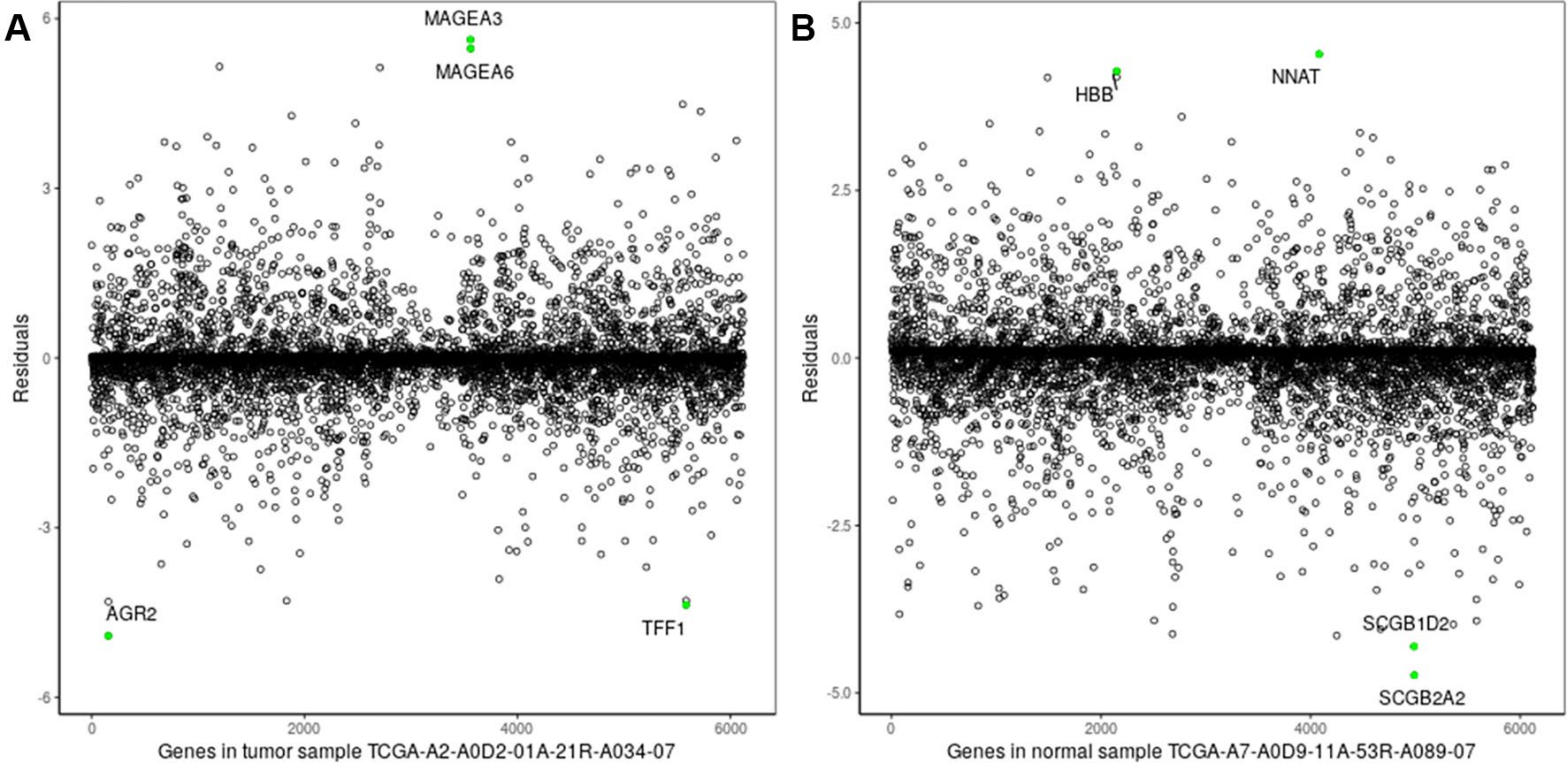
Residuals of genes of a single sample. **(A)** Breast tumor sample TCGA-A2-A0D2-01A-21R-A034-07; **(B)** normal tissue sample: TCGA-A7-A0D9-11A-53R-A089-07. The green points denote the two biomarker genes with the highest/lowest expression levels in the two samples.

**Figure 4:**
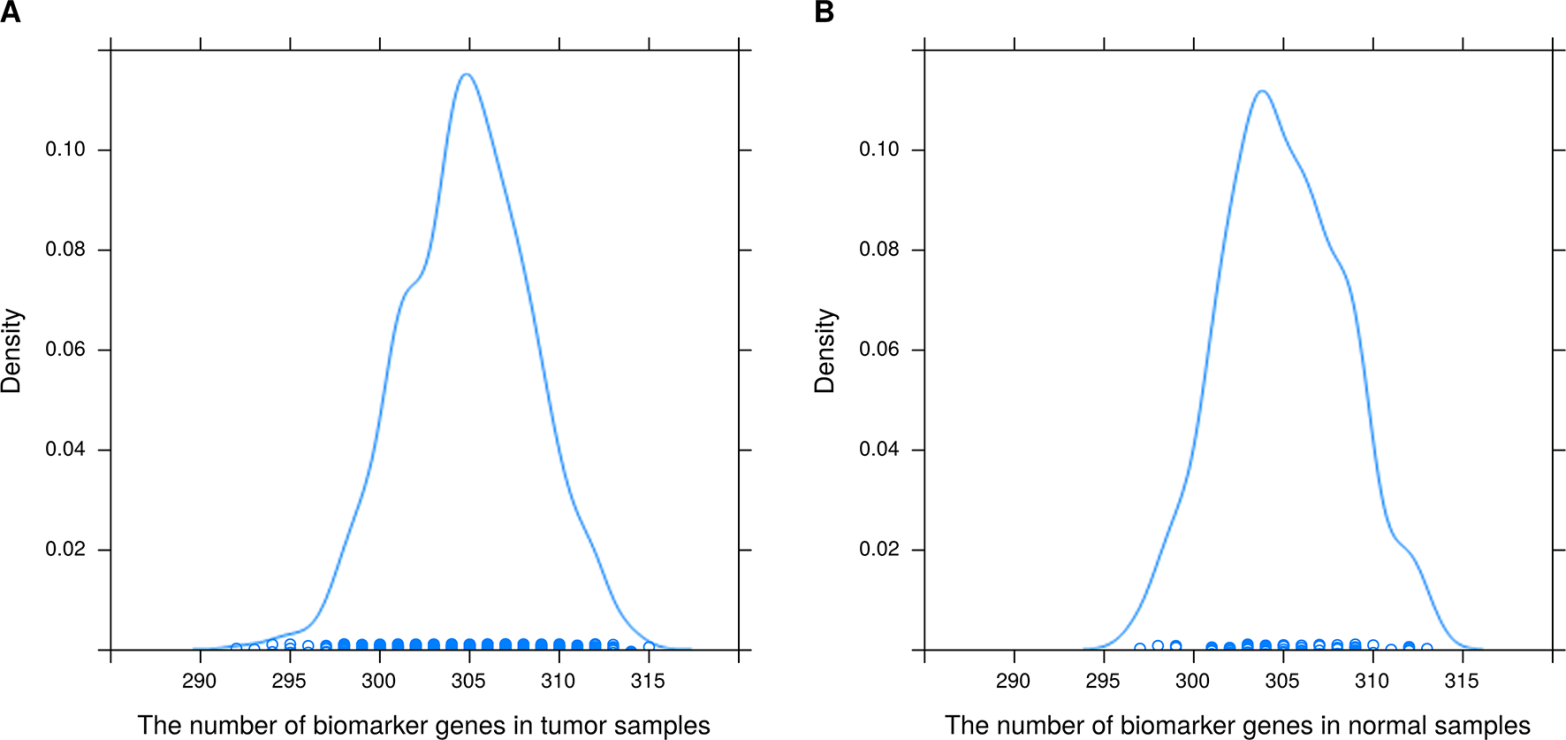
Distribution of the number of biomarker genes in **(A)** 1090 breast tumor samples and **(B)** 113 normal tissue samples.

The experiments on TCGA-BRCA were performed as follows. First, 6120 DEGs in two groups of samples were identified using DESeq2 (Love et al., 2014) at a 95% confidence level and absolute value of log fold change > 1. Next, average tumor and normal samples based on 6120 DEGs were obtained using Equations. (1) and (2). Then, 1,090 (113) regression models were constructed based on average tumor (normal) samples and 1,090 tumor (113 normal) samples, respectively; an example is shown in **Figure 2**. The residuals of the genes of each sample were calculated using Equations (5) and (6); **Figure 3** shows residual values of biomarker genes from two samples. Finally, biomarker genes for each sample were identified using Equations (7), (8), and (9). The distribution of the number of biomarker genes in the 1,090 (113) tumor (normal) samples is shown in **Figure 4**.

As shown clearly in **Figures 2** and **3**, genes were distributed in two main areas. The genes scattered in the upper-left of the plots are those with higher expression levels, whereas genes in the lower-right portion have lower expression values, in the single tumor/normal sample. In **Figure 2**, there are several spots that are distant from the regression lines. These spots represent biomarker genes of the single sample. **Figure 3** shows more clearly which genes had very significant variation in expression. For example, the residuals of *CLEC3A* and *CCNO* were 4.92 and 3.83, respectively, significantly higher than the values for other genes; while the residuals of *HIST3H2A* and *TNNT1* were −3.33 and −2.95, respectively, significantly lower than those of other genes.

It can also be seen from **Figure 4** that the number of biomarker genes varied among different samples. Some tumor samples had more than 315 biomarker genes, while others had about 290. The mean numbers of biomarker genes in the tumor samples and normal samples were 304.9 and 305, respectively. In addition, the biomarker genes of different samples were also different. In 1090 tumor samples and 113 normal samples, the biomarker genes had different frequencies (a biomarker gene has higher frequency if it is found in more samples). The top 15 biomarker genes with significantly different frequencies in tumor and normal samples are listed in **Supplementary Table 1**. These genes were common biomarkers of most tumor samples, and they had higher frequency in tumor samples than in normal samples. Therefore, these genes were likely to be related to the development of breast cancer. To test our hypothesis, we searched the literature using public databases and found that 14 of the 15 genes were indeed related to the development of breast cancer. The top gene was *S100A7*, which has been found to be expressed in several tissues including breast adenocarcinomas and squamous carcinomas of the head and neck, the cervix, and the lung (Emberley et al., 2004); *S100A7* is also related survival of breast cancer patients (Emberley, 2003). *CLEC3A* had the highest frequency in tumor samples; its overexpression promotes tumor progression and poor prognosis in breast invasive ductal cancer (IDC) and is related to higher lymph node and poorer overall survival (OS) of breast IDC (Ni et al., 2018). *PRAME* has a tumor-promoting role in triple-negative breast cancer, increasing cancer cell motility through the epithelial-to-mesenchymal transition (EMT) gene reprogramming. Therefore, *PRAME* could serve as a prognostic biomarker and/or therapeutic target in triple-negative breast cancer (Al-Khadairi et al., 2019). Kammerer et al. (2016) suggested that patients with estrogen receptor-positive breast cancer might be stratified into high- and low-risk groups based on the *KCNJ3* levels in the tumor. *CST1* was found to be generally upregulated in breast cancer at both the mRNA and the protein level. Furthermore, OS and disease-free survival in the low *CST1* expression subgroup were significantly superior to those in the high *CST1* expression subgroup, indicating that *CST1* could be a prognostic indicator and a potential therapeutic target for breast cancer (Dai et al., 2017). Xuan et al. (2015) reported that higher expression of *MMP1* in breast cancer might play a crucial part in promoting breast cancer metastasis. Powell et al. (2018) demonstrated that *CEACAM5* was a clinically relevant driver of breast cancer metastasis. *NKAIN1* is associated with OS in breast cancer (Su et al., 2019). *DSCAM-AS1* promotes tumor growth in breast cancer by reducing miR-204-5p and upregulating *RRM2* (Liang et al., 2019). Overexpression of *CEACAM6* promotes migration and invasion of estrogen-deprived breast cancer cells (Lewis-Wambi et al., 2008). Bhakta et al. (2018) suggested that anti-GFRA1-vcMMAE ADC might provide a targeted therapeutic opportunity for luminal A breast cancer patients. *BMPR1B* is related to proliferation of breast cancer cells (Bokobza et al., 2009). Jia et al. (2016) identified *COL11A1* as a highly specific biomarker of activated cancer-associated fibroblasts (CAFs), which could promote breast cancer and inhibit pancreatic cancer. In summary, 14 of the top 15 biomarker genes have been reported to be associated with breast cancer. Therefore, these results demonstrate that the proposed method can effectively identify biomarkers related to cancer.

Statistical tests were performed to evaluate whether expression levels of biomarker genes of a sample were significantly different compared with those of other samples. As the biomarker gene set of each sample was represented by a p-value vector with dimension n, 1,090*1,089 [n(n−1)], where n is the number of samples) p-values were obtained for the 1090 tumor samples, and 113*112 p-values for the 113 normal samples; 1,186,999 (99.99%) and 12,626 (99.76%) of these p-values were less than 0.05 for the tumor samples and normal samples, respectively. These results indicate that there were significant differences between the expression levels of the identified biomarker genes of a sample and those of other samples, that is, the proposed method can effectively identify the biomarker genes of a single sample.

**Figure 5:**
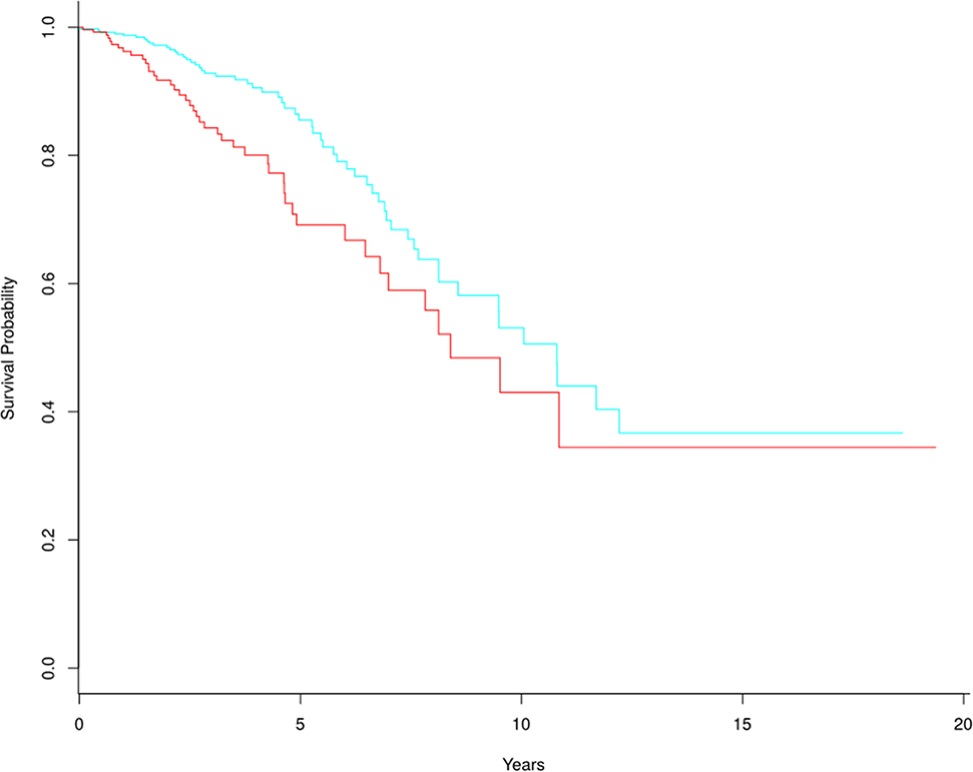
Kaplan-Meier survival curves based on 305 tumor biomarker genes. In the high-risk group (red line), there are 329 tumor samples. In the low-risk group (blue line), there are 759 tumor samples.

The frequencies of biomarker genes in tumor and normal samples were different. Here, we mainly analyzed biomarker genes whose frequency was higher in tumor samples than in normal samples, to explore which genes might have important roles in survival prediction and development of breast cancer. We selected 305 biomarker genes with higher frequency in tumor samples, and clustered the tumor samples into two groups using the multiple survival screening (MSS) algorithm (Li et al., 2010). Survival was significantly different between the two groups (p-value = 0.0089) (**Figure 5**). This means these biomarker genes are important features of breast cancer and can be used to distinguish tumor patients into high- and low-risk groups (here, we removed two samples with the negative follow-up-time, so there were 1,088 samples participating in survival analysis).

### Experimental Results for Immunotherapeutic Response Samples

**Figure 6:**
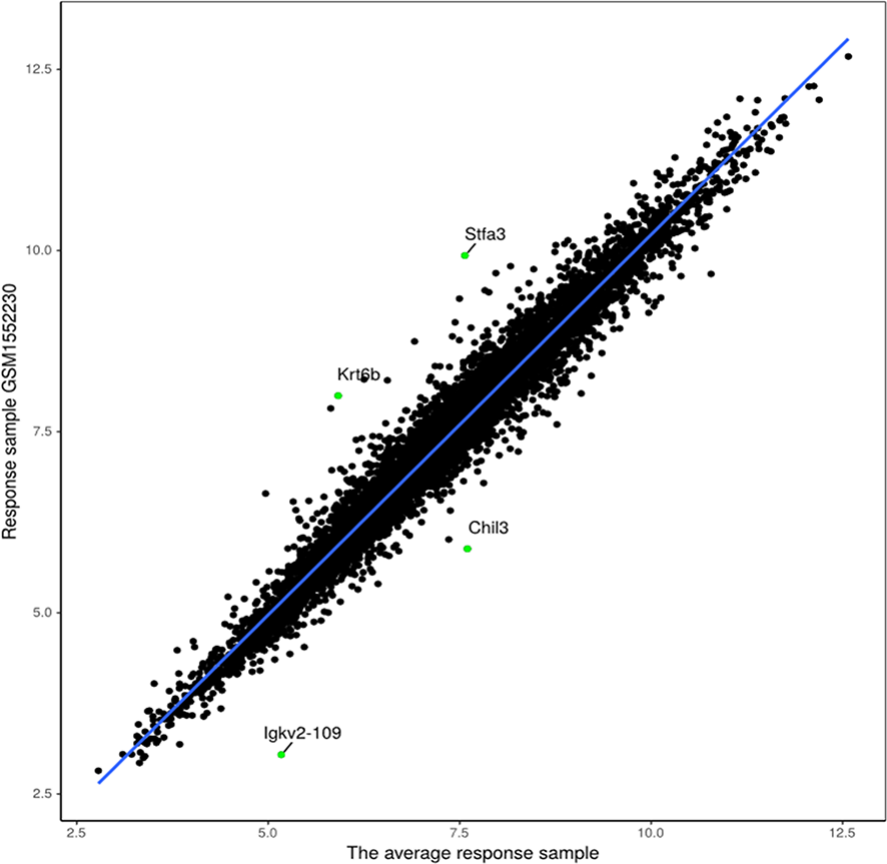
Regression model based on response sample GSM1552230 and the average response sample.

**Figure 7:**
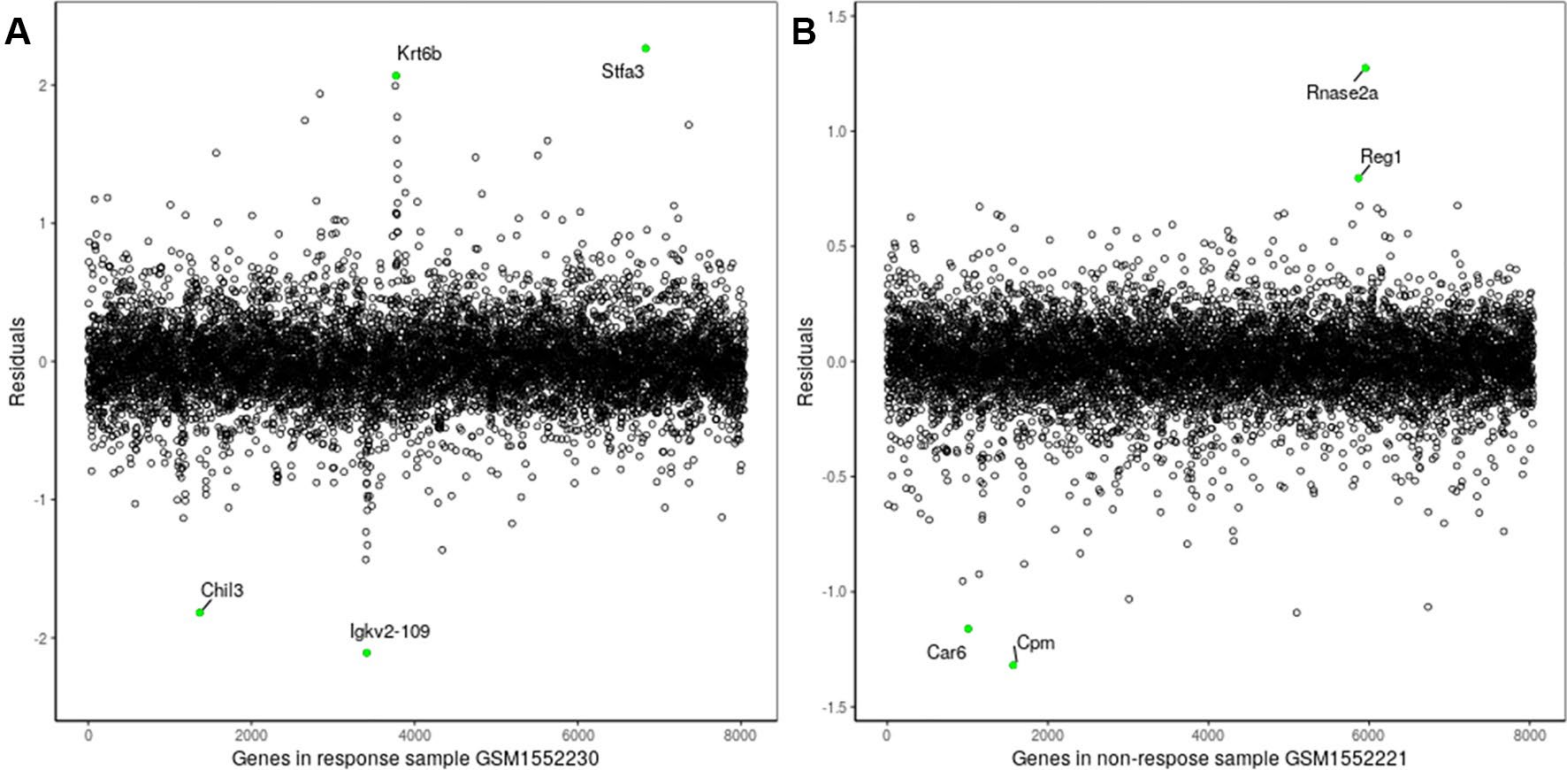
Residuals of biomarker genes **(A)** GSM1552230, **(B)** GSM1552221.

**Figure 8:**
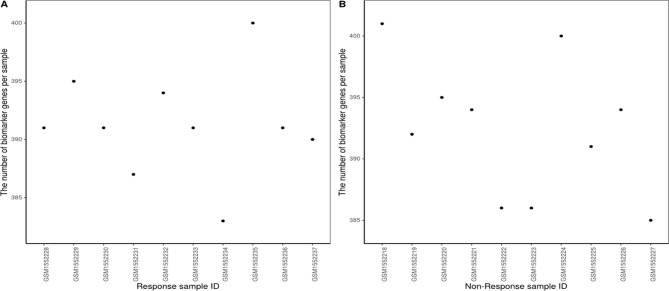
Number of biomarker genes in **(A)** response samples and **(B)** non-response samples.

The proposed method was also used to analyze mouse AB1-HA tumor data: GSE63557. A total of 8,042 DEGs in two groups of samples were identified using GEO2R (Smyth, 2004) at a 95% confidence level. Regression models of 10 anti-CTLA-4 immunotherapeutic response samples and 10 non-response samples were constructed; one of these is shown in **Figure 6**. **Figure 7** shows residual values of biomarker genes from two samples. The number of biomarker genes of 10 response samples and 10 non-response samples is shown in **Figure 8**. In **Figures 6** and **7**, there are several genes that are far from the regression lines. For example, the residuals of *Krt6b* and *Stfa3* were 2.07 and 2.26, respectively, significantly higher than those of other genes; the residuals of *Chil3* and *Igkv2-109* were −1.82 and −2.10, respectively, significantly lower than those of other genes.

The number of biomarker genes of different samples is shown in **Figure 8**, illustrating the variation between samples. The biomarker genes from different samples were also different. For 10 response samples and 10 non-response samples, the top 15 genes with the most significant differences in frequency are shown in **Supplementary Table 2**. Four of these genes, *Gzme*, *CD38*, *CD3D*, and *Chil3*, appeared in the important cancer modules identified by Lesterhuis et al. (2015) However, the top gene, *Jchain*, had not been identified as a member of these important cancer modules; notably, *Jchain* was also found to be the most important of the anti-CTLA-4 immunotherapeutic response biomarker genes in our study, with frequencies in response and non-response samples of 80% and 0%, respectively. This suggests that *Jchain* is related to immunotherapeutic response. GeneCards (https://www.genecards.org/) indeed confirms that *Jchain* has an important role in immune response. Moreover, *Iglj1*, *Cd38*, and *Cd3d* are also immune response related. This demonstrates that the IBI method can detect important genes contributing to the immunotherapeutic response mechanism.

**Figure 9:**
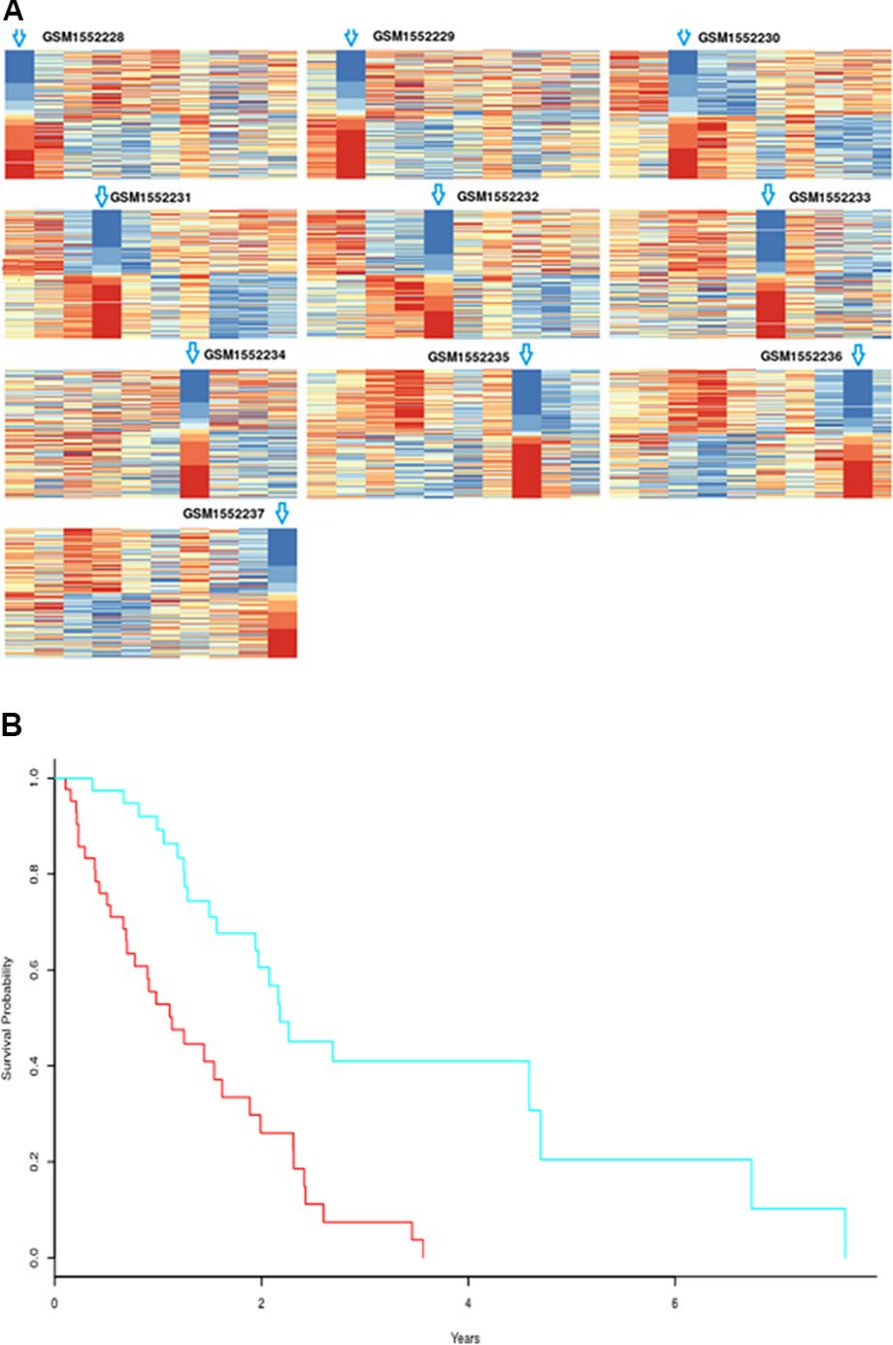
**(A)** Rank matrix of each response sample. **(B)** Kaplan-Meier survival curves for human mesothelioma tumor samples based on biomarker genes from mouse AB1-HA tumor samples; p-value=1.57×10^-5^. High-risk group includes 44 samples; low-risk group consists of 40 samples.

According to the statistical tests, 100% of p-values were less than 0.05 in both response and non-response samples. The rank matrix of each response sample is shown in **Figure 9A**. These results indicate that there are significant differences between the identified response biomarker genes of a sample and those of other samples, that is, the proposed method also can effectively identify biomarker genes of individual samples even when fewer samples are used. We wanted to analyze biomarker genes whose frequency was higher in response samples than in non-response samples, and estimate their ability to predict survival in AB1-HA tumor samples. However, there was no follow-up information for AB1-HA mice. The selected 392 biomarker genes with higher frequency were tested against a human mesothelioma data set (TCGA-MESO, https://portal.gdc.cancer.gov). Notably, these biomarker genes could still effectively distinguish all patients into high- and low-risk groups (**Figure 9B**) with a p-value of 1.57×10^-5^. These results further support the validity of the proposed method.

### Experimental Results for Advanced Melanoma Data

**Figure 10:**
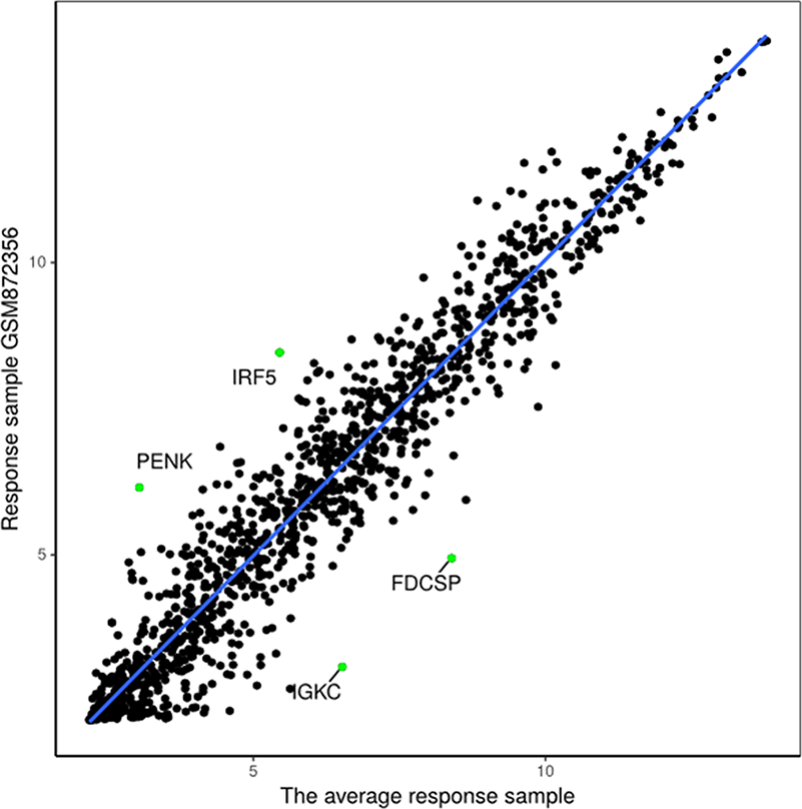
Regression model based on response sample GSM872356 and the average response sample from GSE35640 gene expression data.

**Figure 11:**
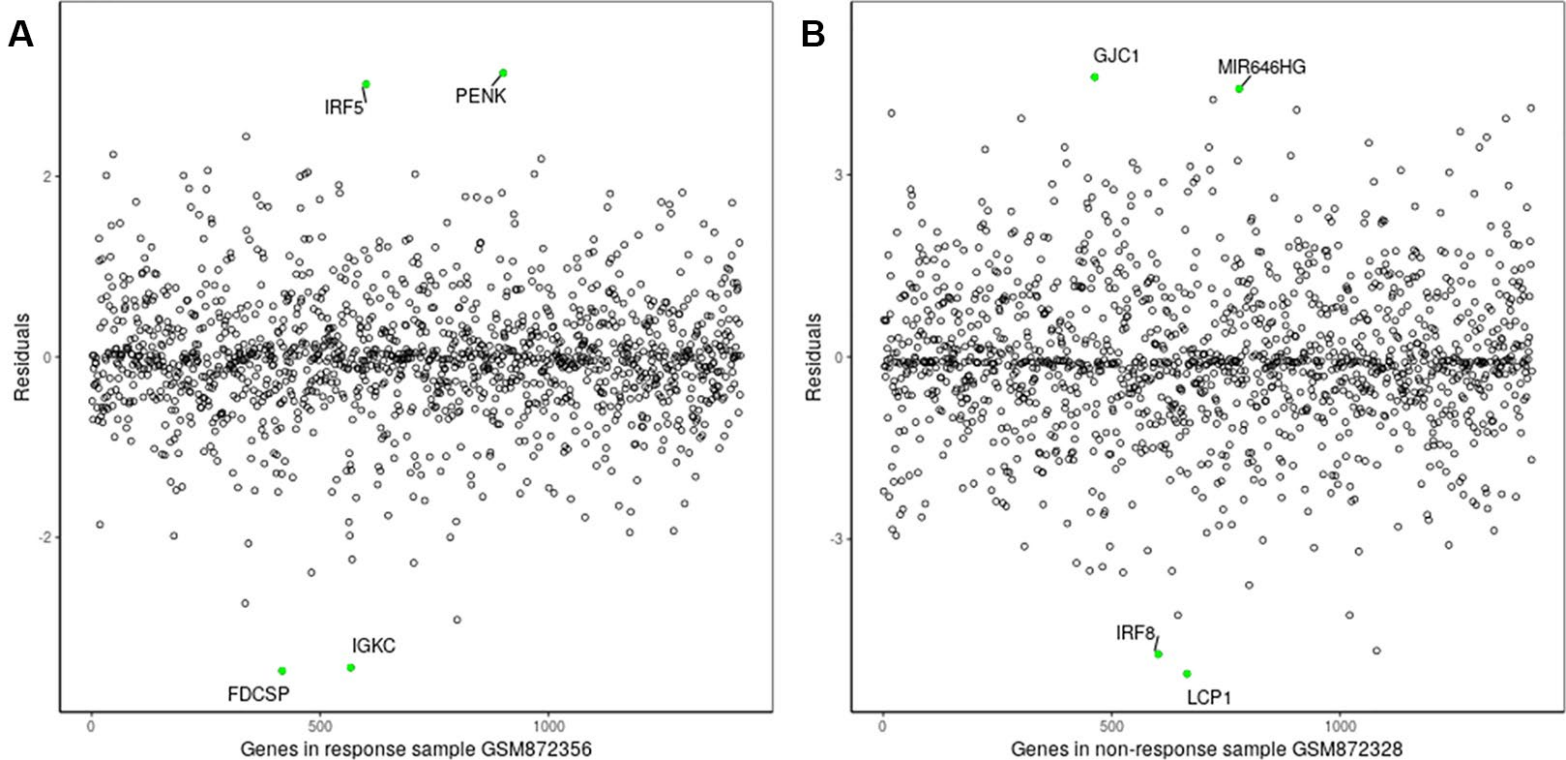
Residuals of biomarker genes. **(A)** GSM872356, **(B)** GSM872328.

**Figure 12:**
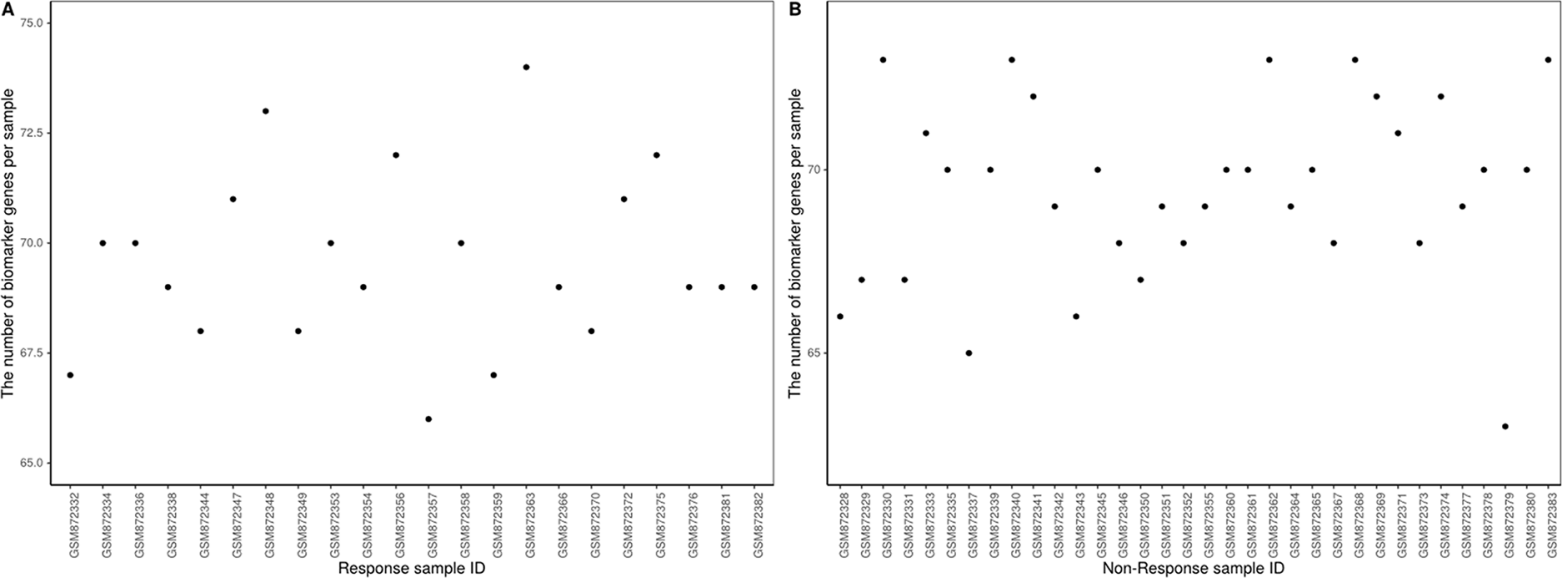
Number of biomarker genes in **(A)** response samples and **(B)** non-response samples.

The proposed method was used to analyze advanced melanoma data: GSE35640. A total of 1420 DEGs were identified in 22 MAGE−A3 immunotherapeutic response and 34 non-response samples using GEO2R (Smyth, 2004) at a 95% confidence level. Regression models of 22 MAGE−A3 immunotherapeutic response and 34 non-response samples were constructed; one of these is shown in **Figure 10**. **Figure 11** shows residual values of biomarker genes from two samples. The number of biomarker genes of 22 response samples and 34 non-response samples is shown in **Figure 12**.

As shown in **Figure 12**, there were small differences in the number of biomarkers from different samples. The mean number of biomarker genes in response samples was 70. The top 15 genes with the most significant difference of frequency in 22 response samples and 34 non-response samples are shown in **Supplementary Table 3**. We proposed that these genes were likely to be mainly immune or tumor related. To test our hypothesis, we searched GeneCards for these genes and found that some of them play important roles in the development of immune-related cells. For example, *MS4A1* is associated with the development of B-cells into plasma cells; *CD37* may play a part in T-cell–B-cell interactions; *CD5L* participates in obesity-associated autoimmunity; *MMP8*, *IRF5*, and *RHOF* are related to innate immune pathways; *MMP9* has a role in tumor-associated tissue remodeling; and *TRAM1L1* is related to the well-known cancer-related NF-kB pathway. This demonstrated that the IBI method could detect important genes contributing drug response mechanisms and help to elucidate immunotherapeutic response mechanisms. In the statistical tests, 96.96 and 95.72% of p-values were less than 0.05 in the response and non-response samples, respectively. These results also indicate that biomarker genes of a sample show significant differences compared with those of other samples, that is, the proposed method can also effectively identify MAGE−A3 immunotherapeutic response biomarker genes in individual advanced melanoma samples even with fewer samples.

**Figure 13:**
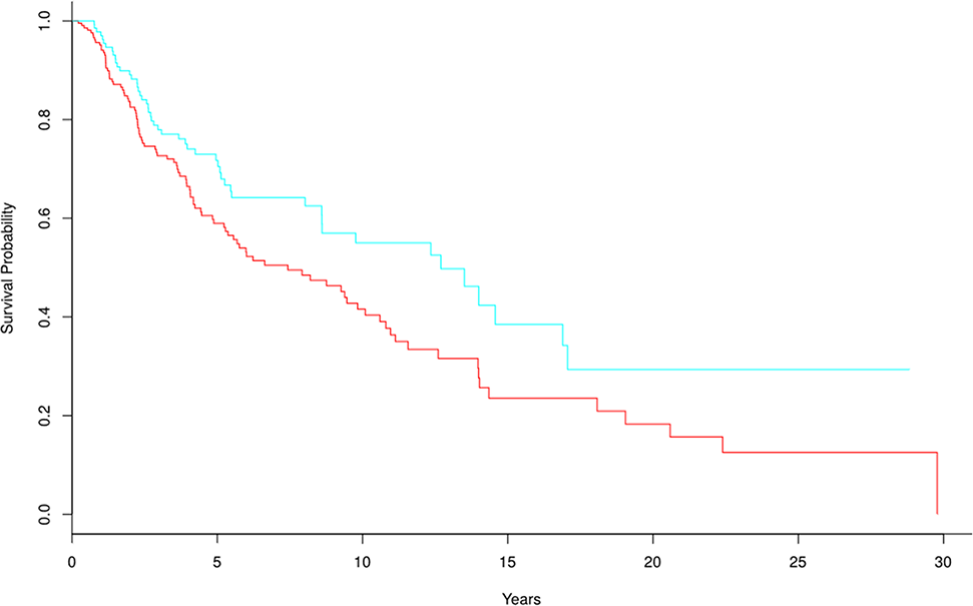
Kaplan-Meier survival curves for TCGA-SKCM based on biomarker genes from GSE35640; p-value = 0.016. There were 281 and 166 samples in the high-risk and low-risk groups, respectively.

We wanted to analyze biomarker genes whose frequency was higher in response samples than in non-response samples, and estimate their ability to predict survival in advanced melanoma. However, there was no follow-up information in GSE35640, so we used skin cutaneous melanoma gene expression data (TCGA-SKCM) for the survival analysis. The selected 70 biomarker genes were tested against TCGA-SKCM, showing that these biomarker genes could effectively distinguish skin cutaneous melanoma patients into high- and low-risk groups (**Figure 13**), with a p-value of 0.016. These results indicate that the proposed method performs well. In their original paper, Ulloa-Montoya et al. (2013) identified 84 gene expression signatures associated with response to MAGE-A3 immunotherapy in metastatic melanoma and non-small-cell lung cancer, whereas 61 of the 84 genes were chosen as biomarker genes by our proposed method (e.g., *CD86*, *CCL5*, and *IRF1*). These genes were mainly immune related and were involved in interferon gamma pathways and specific chemokines. Experimental results showed that pretreatment MAGE-A3 immunotherapy in metastatic melanoma influenced the tumor’s immune microenvironment and the patient’s clinical response. The proposed method could be used to identify these biomarker genes and predict the influence of MAGE-A3 immunotherapy on survival in metastatic melanoma (**Figure 13**).

### Experimental Results for the Simulated Data

In order to further test the performance of the proposed method, we added a supplemental experiment on the simulated gene expression data. First, the simulated gene expression data with 10 samples 1000 genes is generated using *simulateGEdata* function in the *RUVcorr* (Freytag et al., 2015) package. Then, 1,000 genes are divided into 10 groups, we increase/decrease gene expression value of the *i*th group of genes in the *i*th sample by an up or down perturbation value. The range of perturbation value is from 0 to mean value of the corresponding gene in 10 samples. Thus, the *i*th group of genes can be considered as biomarker genes of the *i*th sample. Finally, experiment is performed on the simulated data to observe whether the proposed method can find these markers. We repeated the above steps ten times and experimental results shown that the proposed method can effectively identify the biomarker genes of 10 samples. The 99% biomarker genes identified by the proposed method are the predefined biomarkers when the perturbation value is twice (see **Supplementary Figure 1**).

## Discussion

Precision medicine is an active area of cancer research. The key to cancer precision medicine is to find biomarker genes with high performance, and various approaches to identify such genes have been developed. However, identification of biomarker genes for individual tumor samples remains a challenging problem; for many reasons, there is a lack of effective approaches to identify biomarkers in individual patients. Here, we developed a novel approach to address this issue. Experimental results based on several different data sets show that the proposed method can effectively identify biomarker genes of individual human tumor samples, not only from several hundred samples but also from a few samples without clinical information, and even from mouse samples.

## Data Availability Statement

Publicly available datasets were analyzed in this study: TCGA-BRCA data (found at The Cancer Genome Atlas), GSE63557 (found at Gene Expression Omnibus) and GSE35640 (found at Gene Expression Omnibus).

## Author Contributions

JL and DW designed and implemented the algorithm. JL and DW analyzed the results and wrote the manuscript, and YW made suggestions. All authors read and approved the final manuscript.

## Funding

This work was partially supported by National Key Research and Development Program of China (Grant No.2016YFC0901905) and the Natural Science Foundation of Heilongjiang Province (Grant No. F2016016).

## Conflict of Interest

The authors declare that the research was conducted in the absence of any commercial or financial relationships that could be construed as a potential conflict of interest.
