# Supplementary material for: IBI: Identification of Biomarker Genes in Individual Tumor Samples
Source: Front Genet. 2019 Nov 26;10:1236. doi: 10.3389/fgene.2019.01236 (PMC6902017; doi:10.3389/fgene.2019.01236)
Supplement: Supplementary file 1 [file DataSheet_1.docx]

Supplementary Material

# Supplementary Tables

Supplementary Table 1. Top 15 biomarker genes with significant different frequency in breast tumor and normal samples

| ***Order*** | ***GS ^a^*** | ***F ^b^*** | ***F ^c^*** | ***Difference*** |
| --- | --- | --- | --- | --- |
| 1 | S100A7 | 0.76 | 0.09 | 0.68 |
| 2 | CLEC3A | 0.85 | 0.21 | 0.64 |
| 3 | PRAME | 0.65 | 0.03 | 0.62 |
| 4 | KCNJ3 | 0.64 | 0.04 | 0.59 |
| 5 | SYT13 | 0.60 | 0.02 | 0.58 |
| 6 | CST1 | 0.59 | 0.02 | 0.58 |
| 7 | MMP1 | 0.56 | 0.02 | 0.54 |
| 8 | CEACAM5 | 0.57 | 0.04 | 0.52 |
| 9 | NKAIN1 | 0.60 | 0.08 | 0.52 |
| 10 | DSCAM-AS1 | 0.54 | 0.03 | 0.51 |
| 11 | CEACAM6 | 0.70 | 0.19 | 0.51 |
| 12 | GFRA1 | 0.65 | 0.15 | 0.50 |
| 13 | BMPR1B | 0.76 | 0.26 | 0.50 |
| 14 | CYP2B7P | 0.73 | 0.24 | 0.49 |
| 15 | COL11A1 | 0.49 | 0.01 | 0.48 |

^a^ Gene Symbol, ^b^ Frequency in tumor samples, ^c^ Frequency in normal samples

Supplementary Table 2. Top 15 biomarker genes with significant different frequency in response and non-response samples from mice AB1-HA tumor data

| Order | GS ^a^ | F ^b^ | F ^c^ | Difference |
| --- | --- | --- | --- | --- |
| 1 | Jchain | 0.8 | 0 | 0.8 |
| 2 | Iglj1 | 0.9 | 0.2 | 0.7 |
| 3 | Mospd1 | 0.7 | 0 | 0.7 |
| 4 | Trav7-1 | 0.7 | 0 | 0.7 |
| 5 | Ace2 | 0.9 | 0.3 | 0.6 |
| 6 | Gzme | 0.8 | 0.2 | 0.6 |
| 7 | 1700061E17Rik | 0.6 | 0 | 0.6 |
| 8 | 2410141K09Rik | 0.7 | 0.1 | 0.6 |
| 9 | Casp14 | 0.6 | 0 | 0.6 |
| 10 | Cd38 | 0.6 | 0 | 0.6 |
| 11 | Cd3d | 0.6 | 0 | 0.6 |
| 12 | Gk2 | 0.7 | 0.1 | 0.6 |
| 13 | Igkv6-32 | 0.6 | 0 | 0.6 |
| 14 | Xlr | 0.6 | 0 | 0.6 |
| 15 | Chil3 | 0.8 | 0.3 | 0.5 |

^a^ Gene Symbol, ^b^ Frequency in response samples, ^c^ Frequency in non-response samples

Supplementary Table 3. Top 15 biomarker genes with significant different frequency in response and non-response samples from advanced melanoma data

| ***Order*** | ***GS ^a^*** | ***F ^b^*** | ***F ^c^*** | ***Difference*** |
| --- | --- | --- | --- | --- |
| 1 | MMP9 | 0.59 | 0.03 | 0.56 |
| 2 | LOC101929450 | 0.68 | 0.15 | 0.53 |
| 3 | LRP2 | 0.68 | 0.15 | 0.53 |
| 4 | IGLL5 | 0.59 | 0.12 | 0.47 |
| 5 | MS4A1 | 0.59 | 0.24 | 0.36 |
| 6 | IGHD | 0.41 | 0.06 | 0.35 |
| 7 | RHOF | 0.41 | 0.09 | 0.32 |
| 8 | CPS1 | 0.45 | 0.15 | 0.31 |
| 9 | ELOVL6 | 0.41 | 0.12 | 0.29 |
| 10 | IRF5 | 0.41 | 0.12 | 0.29 |
| 11 | TRAM1L1 | 0.41 | 0.12 | 0.29 |
| 12 | CD37 | 0.32 | 0.06 | 0.26 |
| 13 | CD5L | 0.32 | 0.06 | 0.26 |
| 14 | HLA-DRB4 | 0.32 | 0.06 | 0.26 |
| 15 | MMP8 | 0.32 | 0.06 | 0.26 |

^a^ Gene Symbol, ^b^ Frequency in response samples, ^c^ Frequency in non-response samples


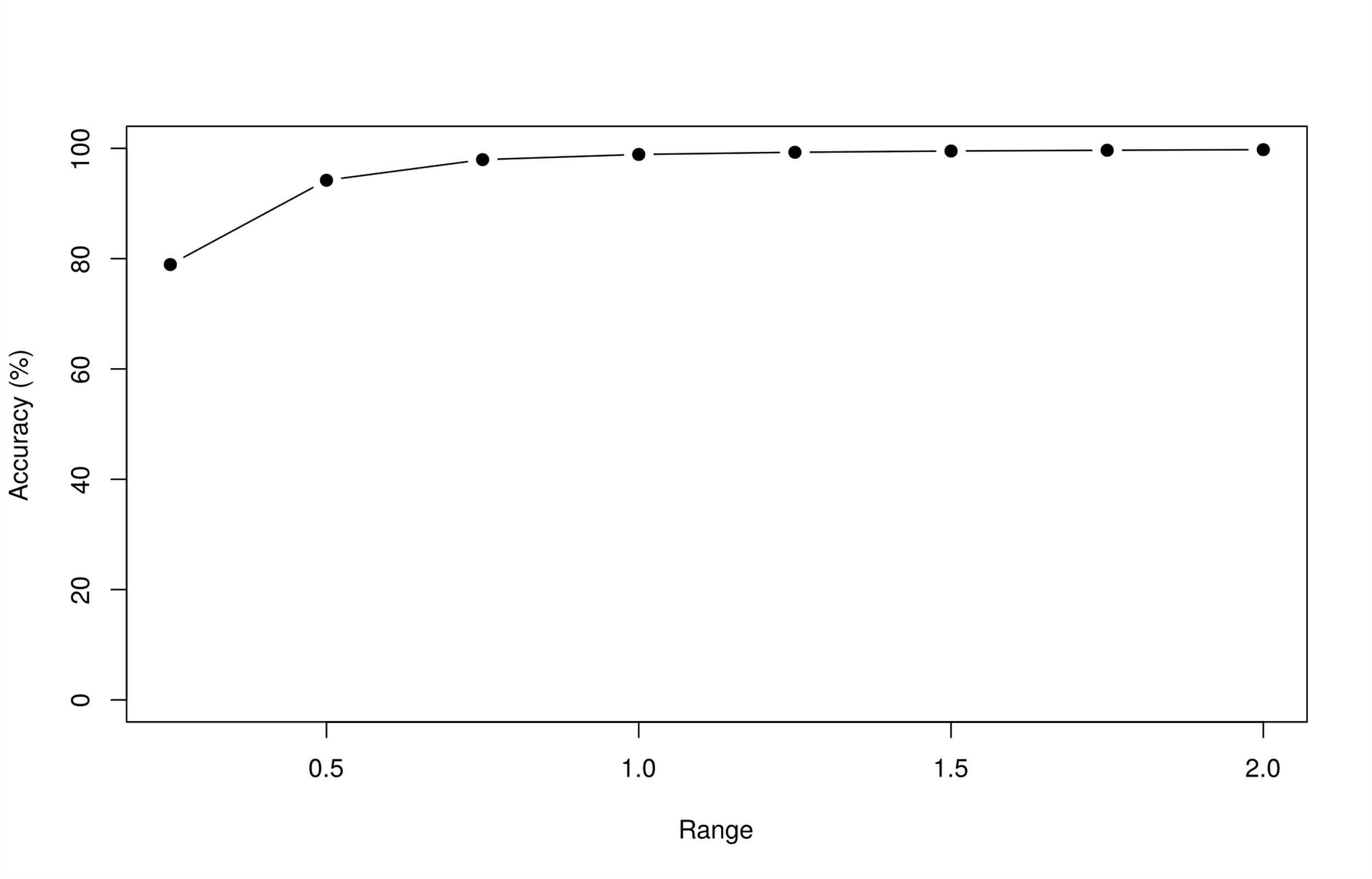


Supplementary Figure 1. The accuracy of the proposed method on simulated data. Range is the ratio of the perturbation value to the corresponding gene average expression value.
